# Can Pain Neuroscience Education Combined with Cognition-Targeted Exercise Therapy Change White Matter Structure in People with Chronic Spinal Pain? A Randomized Controlled Trial

**DOI:** 10.3390/jcm14030867

**Published:** 2025-01-28

**Authors:** Iris Coppieters, Jo Nijs, Mira Meeus, Lieven Danneels, Nathalie Roussel, Barbara Cagnie, Jeroen Kregel, Ward Willaert, Emma Rheel, Robby De Pauw, Anneleen Malfliet

**Affiliations:** 1 Pain in Motion Research Group (PAIN), Department of Physiotherapy, Human Physiology and Anatomy (KIMA), Faculty of Physical Education & Physiotherapy, Vrije Universiteit Brussel, Laarbeeklaan 103—Building F, 1090 Brussels, Belgium; 2 Experimental Health Psychology Research Group, Faculty of Psychology and Neuroscience, Maastricht University, 6211 LK Maastricht, The Netherlands; 3 The Laboratory for Brain-Gut Axis Studies (LaBGAS), Translational Research Center for Gastrointestinal Disorders (TARGID), KU Leuven, 3000 Leuven, Belgium; 4 Department of Physical Medicine and Physiotherapy, University Hospital Brussels, Laarbeeklaan 101, 1090 Brussels, Belgium; 5 Department of Rehabilitation Sciences and Physiotherapy (MOVANT), Faculty of Medicine and Health Sciences, University of Antwerp, Campus Drie Eiken, Universiteitsplein 1, 2610 Wilrijk, Belgium; 6 Department of Rehabilitation Sciences, Faculty of Medicine and Health Sciences, Ghent University, Campus UZ Gent, Building B3, Corneel Heymanslaan 10, 9000 Ghent, Belgium; 7 Breederode Hogeschool, 3011 Rotterdam, The Netherlands; 8 Research Foundation—Flanders (FWO), 1000 Brussels, Belgium

**Keywords:** central sensitization, chronic pain, diffusion tensor imaging, neurorehabilitation, neuroplasticity, white matter

## Abstract

**Background/Objectives:** White matter (WM) structural changes have been found in patients with chronic spinal pain (CSP). In these patients, pain neuroscience education followed by cognition-targeted exercise therapy (i.e., the Modern Pain Neuroscience Approach (MPNA)) was shown to be more effective than biomedically-focused education followed by symptom-contingent exercise therapy for improving clinical outcomes. The present study examined whether an MPNA, compared to biomedically-focused treatment, can change WM structure in regions of interest and whether potential WM structural changes are associated with clinical improvements in patients with CSP. **Methods**: Patients with CSP were randomized into an experimental (MPNA) or control (biomedically-focused) treatment group. Diffusion-weighted Magnetic Resonance Images were acquired pre-treatment, post-treatment, and at 1-year follow-up. WM structure was assessed using diffusion tensor imaging in 8 WM regions of interest, and linear mixed models assessed differences between groups in response to treatment. **Results**: No significant treatment x time interaction effects were found; however, significant main effects of time were found in 7 WM tracts. Significant main effects of time revealed increased fractional anisotropy (FA), decreased mean diffusivity (MD), axial diffusivity (AD), and radial diffusivity (RD) in the cingulum hippocampus, and decreased RD and MD in the superior cerebellar peduncle at 1-year follow-up compared to baseline. In contrast, decreased FA and/or increased MD, AD, or RD values were found in other WM tracts (e.g., anterior corona radiata) from pre-treatment to 1-year follow-up. Greater reduction in kinesiophobia was moderately correlated with a smaller decrease in RD in the superior cerebellar peduncle at 1-year follow-up compared to baseline. No other significant associations were found between WM structural changes and clinical improvements. **Conclusions**: In conclusion, in patients with CSP, regional WM structure changed over time irrespective of prescribed treatment (timespan of 12 months). Further research, including Neurite Orientation Dispersion and Density Imaging and a healthy control group, allowing for a more specific examination of WM microstructural changes in response to multimodal treatment in patients with CSP, is warranted.

## 1. Introduction

Chronic spinal pain (CSP) includes chronic neck pain (CNP) and chronic low back pain (CLBP) and is a prevalent global problem that leads to disability and interferes with patients’ daily activities [1,2]. CSP is multifactorial and influenced by a range of psychosocial [3,4], neurophysiological [5,6], and pathoanatomical [7] factors.

Studies discovered that brain structural (i.e., white matter (WM) or grey matter) and functional changes and central sensitization (i.e., amplification of neural signaling within the central nervous system that elicits pain hypersensitivity [8]) can play a key role in chronic pain-related disability in individuals with chronic pain [6,9,10,11] and therefore may be important treatment targets. Diffusion tensor imaging (DTI) allows us to visualize and assess the direction and degree of water diffusivity in WM tracts [12]. More specifically, the degree of fractional anisotropy (FA) of water in the WM tracts reflects their level of integrity [13]. Human brain imaging studies have suggested that a decrease in brain WM integrity (examined using FA) can be associated with damage (e.g., traumatic brain injury) [14] or degenerative conditions (e.g., stroke or increasing age) [15,16]. Interestingly, patients with CSP studies also showed lower FA values in comparison to healthy pain-free people [17,18,19,20]. Moreover, some of these studies found that lower FA values coincided with higher values of mean and radial diffusivity (MD and RD, respectively), which are related to structural disintegration [18,19]. Importantly, these WM changes show a positive association with pain severity and pain catastrophizing [20]. Therefore, a treatment that targets pain severity and pain cognitions (including pain catastrophizing) might be able to alter WM structure.

Such a treatment comprising pain neuroscience education followed by cognition-targeted exercise therapy (i.e., Modern Pain Neuroscience Approach (MPNA)) has been found to be effective in improving pain, disability, function, and pain cognitions in people with CSP [21,22]. This approach begins by educating the patient on the mechanisms underlying chronic pain, followed by the use of time-contingent functional exercises to enable the patient to progressively engage in movements and activities they previously feared [21,23]. While the clinical effectiveness of this approach was established, its effect on WM structure remains unstudied. Moreover, despite the prevalence of WM structural changes in patients with CSP [18,19,24,25], there is in general a lack of studies examining WM changes in response to an effective chronic pain treatment.

Therefore, to fill this knowledge gap, this study aims to investigate (1) whether the MPNA is, besides being clinically effective, also an effective treatment to change WM structure in regions of interest compared to biomedically-focused education followed by symptom-contingent exercise therapy (biomedically-focused treatment) in patients with nonspecific CSP, and (2) whether changes in WM structure are associated with improvement in various clinical outcomes. We hypothesize that the MPNA is more effective for altering the WM structure compared to usual care evidence-based physiotherapy because the MPNA specifically targets the brain, and we assume that WM changes will be associated with clinical improvements.

## 2. Materials and Methods

### 2.1. Trial Design

This study was carried out as a Randomized Controlled Trial (RCT) and was ethically approved by the ethical committees of the University Hospitals of Ghent (ID nr. 2013/1133) and Brussels (ID nr. 2013/385). This study was carried out between January 2014 and January 2017. All participants provided written informed consent prior to enrolment. The full protocol is registered online (clinicaltrials.gov: ID: NCT02098005, 17 March 2014). The main study results, including treatment effects on pain, function, pain cognition, and brain grey matter morphology, are published elsewhere [22]. Results on WM structure have not been reported before, and WM structure was a secondary outcome of the RCT.

### 2.2. Study Population

Participants were recruited through flyers in the University Hospitals and Universities, occupational health services, primary care practices, social media, and advertisements. Based on the inclusion criteria, eligible patients were enrolled to participate in this study.

Inclusion criteria included native Dutch-speaking male and female patients aged between 18 and 65 years, having nonspecific CSP (i.e., CLBP, failed back surgery syndrome more than three years ago, chronic whiplash, or chronic non-traumatic neck pain) at least three days/week for at least three months. Participants were asked not to continue any other therapies during study participation except for their usual medication. Participants were not allowed to start a new therapy or new medication six weeks before and during study participation. Key exclusion criteria were neuropathic pain, a history of neck/back surgery in the past three years, osteoporotic vertebral fractures, rheumatologic diseases, and chronic widespread pain syndromes (i.e., fibromyalgia, chronic fatigue syndrome). People living more than 50 km away from the hospital were excluded to avoid dropout. A total of 120 patients with nonspecific CSP were included in the trial.

### 2.3. Randomization

Randomization was performed at the Biostatistics Unit (Ghent University) by an independent investigator with SAS (9.4) software, using a stratified permuted block allocation (block size of four). Stratification factors were treatment center, dominant pain location, and sex [26].

### 2.4. Experimental Procedures

Participants underwent baseline testing, including brain Magnetic Resonance Imaging (MRI) within 2 weeks before the treatment started. Pressure Pain Thresholds (PPTs) assessments were conducted on a different test day. The following demographic data were collected: age, weight, height, sex, pain duration, dominant pain problem, educational level, and handedness. Additionally, participants were asked to fill out various questionnaires online two weeks before the start of the treatment. Three time-points of assessments included baseline, directly post-treatment, and 1-year post-baseline. All assessments were organized at the same place and were carried out by the same well-trained assessors.

### 2.5. Clinical Outcomes: Pain, Central Sensitization-Related Symptoms, Pain Cognitions, Pressure Pain Sensitivity, and Disability

Clinical outcomes were measured using reliable Dutch-translated questionnaires and PPTs were measured. The detailed information was published elsewhere [22,27].

The Numeric Rating Scale (NRS) measures participants’ spinal pain over the last three days on an 11-point rating scale ranging from 0 (no pain) to 10 (worst pain). A 30% decrease in the NRS score is considered clinically relevant [28]. The Central Sensitization Inventory (CSI) is a patient-reported outcome and assesses somatic and emotional symptoms related to central sensitization in 25 statements related to current health symptoms. The presence of each of these items is measured on a 5-point Likert scale ranging from 0 (never) to 4 (always). A cumulative score ranges from 0 to 100 and higher scores indicate higher symptom frequency/severity [29]. The Pain Catastrophizing Scale (PCS) consists of 13 items scored on a five-point Likert scale and measures aspects of catastrophic cognitions about pain-rumination that individuals may have when experiencing pain. Higher scores indicate more severe catastrophic thoughts about pain [30,31]. The Tampa Scale for Kinesiophobia (TSK) contains 17 items related to fear of movement and re-injury. The score ranges from 17 to 68 (scores ≤ 37 present a low level of fear of movement and scores > 37 present a high level of fear of movement) [32,33]. The Pressure Pain Threshold (PPT) is defined as the point of minimum pressure required to induce an unpleasant sensation. PPTs were measured using a digital algometer (Wagner Instruments, Greenwich, CT) at the most painful side or at the dominant side in case of bilateral pain. PPTs were randomly measured twice at the symptomatic levels for neck pain (NP) (the upper trapezius muscle midway between C7 and the tip of the acromion) and for low back pain (LBP) (5 cm lateral of the spinous process of L3) and at a remote site (quadriceps muscle) [34,35]. At each of the selected measuring points, the threshold was determined as the mean of two consecutive (30 s in between) measurements [34,35]. The Pain Disability Index (PDI) assesses disability associated with pain in social roles (family/household responsibilities, recreation, social activities, occupation, sexual behavior, self-care, and life support activities). The index includes seven items to be scored on an 11-point NRS (0 = no disability and 10 = total disability). The sum score was used in this study (higher scores indicate more disability) [36]. Finally, the Short Form 36 Health Status Survey (SF-36) assesses the functional status and state of well-being and health-related quality of life and results in two main subscales: physical health (SFPH) and mental health (SFMH). The values range from 0 to 100 for each subscale, with an improvement being reflected by an increase in scores.

### 2.6. MRI Data Acquisition

MR images of the brain were acquired on a 3T Siemens Magnetom TrioTim MRI scanner with a 32-channel head coil, located at Ghent Institute for Functional and Metabolic Imaging (GIfMI), Ghent University Hospital, Ghent, Belgium. First, high-resolution T1-weighted MR images were acquired using a three-dimensional magnetization prepared rapid acquisition gradient echo (MP-RAGE) with the following parameters, FoV-matrix = 256 × 256 mm, repetition time (TR) = 2250 ms, echo time (TE) = 4.18 ms, voxel size = 1 × 1 × 1 mm^3^, flip angle = 9°, 176 coronal slices, acquisition time (TA) = 5′14″. All T1-weighted scans were checked by a radiologist to ensure that no brain lesions were present.

Second, diffusion-weighted images of the brain were acquired using single-shot echo planar imaging with a twice-refocused spin echo sequence with FoV = 240 × 240 mm, TR = 10.800 ms, TE = 83 ms, and 60 contiguous sagittal slices (slice thickness = 2.5 mm, voxel size = 2.5 × 2.5 × 2.5 mm^3^), and an acquisition time of 12′36″. Diffusion-sensitizing gradients were applied at a b value of 1200 s/mm^2^, along 64 noncollinear directions. Additionally, one set of images with no diffusion weighting (b-value = 0 s/mm^2^) was acquired as a reference image.

#### 2.6.1. Diffusion-Weighted Imaging Processing

The diffusion-weighted imaging data were analyzed and processed in ExploreDTI v4.8.6 with MATLAB vR2017b (MathWorks, Natick, MA, USA) [37] using the following procedure: diffusion-weighted imaging volumes were looped at a high frame rate to check for obvious artifacts in the data, such as large signal dropouts and geometric distortions. Next, we toggled between the views of the first and last acquired diffusion-weighted images to observe subtle system drifts. Furthermore, we inspected the images in different orthogonal views (coronal, sagittal, axial) to check for interslice and intravolume instabilities, and visualized various image maps to check for artifacts. Then, we checked the residual map for outliers, reflecting the difference between the modeled and the measured signal [12]. Next, the diffusion-weighted data were corrected for distortions induced by the diffusion-weighted gradients, artifacts due to head motion, eddy current-induced geometric distortions, and Echo-planar imaging (EPI) distortions [38]. EPI distortions were corrected by co-registering the diffusion-weighted images to the T1-weighted anatomical images, which were normalized for intensity using FreeSurfer v6.0. Moreover, we performed an appropriate reorientation of the encoding vectors. Next, a tensor estimation procedure was performed on the preprocessed diffusion-weighted data.

Afterward, each scan was visually checked for accuracy after both the motion correction and co-registration steps. To check the accuracy of the co-registration, the preprocessed diffusion-weighted image was overlaid on the normalized T1-weighted anatomical image. We re-inspected the data in three orthogonal planes to ensure that the motion/distortion correction was performed correctly and that no additional artifacts were introduced into the data. Finally, diffusion-weighted images derived metrics FA, MD, axial diffusivity (AD), and RD were extracted from the preprocessed diffusion-weighted imaging data using an automated approach based on the ICBM DTI-81 WM atlas [39].

#### 2.6.2. White Matter Regions of Interest

The ICBM-DTI-81 WM labels atlas, developed by Mori et al. (2008) [39], was used for automated parcellation of the regions of interest (ROIs). This is a stereotaxic probabilistic WM atlas that fuses diffusion tensor imaging-based WM information with the standard MNI anatomical template (ICBM-152). The WM parcellations were applied to the preprocessed images, and the diffusion-weighted image-derived metrics were calculated in each ROI by warping the atlas template to each individual data set.

**Figure 1:**
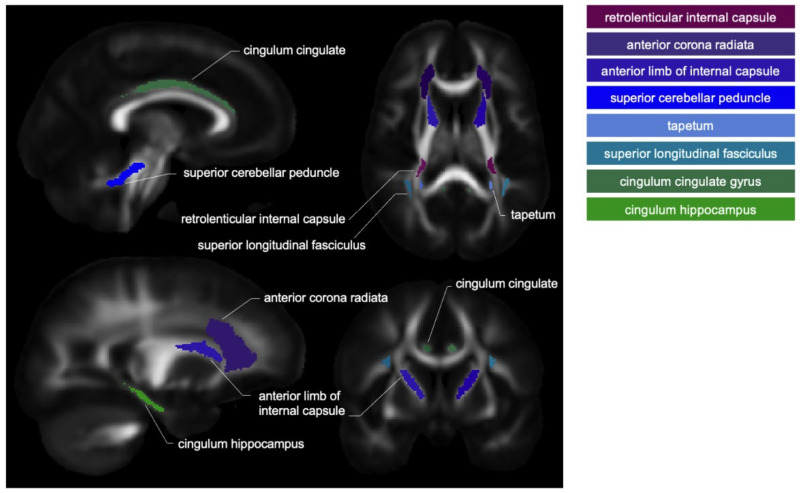
White matter (WM) regions/tracts of interest. WM regions are depicted on mean fractional anisotropy maps.

The selection of WM ROIs was based on previous MRI research demonstrating WM structural changes in patients with CSP. Based on our anatomical hypotheses, only WM atlas labels that are mainly involved in pain were selected. Hereby, the following WM regions/tracts were defined: anterior corona radiata [19], anterior limb of internal capsule [19,25], cingulum (cingulate gyrus) [24], cingulum hippocampus [18], retrolenticular part of the internal capsule [19], superior cerebellar peduncle [40], superior longitudinal fasciculus [19], and tapetum [18] (Figure 1).

To examine WM structural organization, average FA, MD, AD, and RD values were computed in all WM ROIs for each subject for the right and left hemispheres separately.

### 2.7. Interventions

During the 12-week intervention, both experimental and control groups received 18 treatment sessions. According to the protocol, both groups received three educational sessions (group session, home-based online module, and individual session) over the first 2 weeks, followed by 15 individual exercise therapy sessions over the next 10 weeks. Therefore, both interventions were identical in practical organization but substantially different regarding content.

#### 2.7.1. Experimental Group Intervention Including MPNA

The experimental intervention combined three sessions of pain neuroscience education with 15 sessions of cognition-targeted exercise therapy. Pain neuroscience education aims to reframe the patient’s unhelpful and negative beliefs about pain by providing the patient with information about the nature of pain. This approach is known to reduce fear avoidance and avoidance behavior and to increase self-efficacy [21]. Pain neuroscience education includes the following important points in nontechnical terms: the neuron, the synapse, descending nociceptive inhibition and facilitation, peripheral sensitization, and central sensitization using verbal instructions, diagrams, and free-hand drawings [22,23]. Once adaptive beliefs about pain were acquired, cognition-targeted exercise therapy was initiated (session 4). Exercises started with sensorimotor control training by facilitating the proprioceptive system and optimizing the coordinative muscle recruitment patterns. Simultaneously, feared and stressful dynamic and functional exercises were initiated using a graded approach, using an intermediate phase of motor imagery if the level of fear was too high. By the end of the program, participants were exposed to physically demanding tasks and feared movements or activities and were exercising during cognitively and psychosocially stressful conditions [22,23]. Participants were asked to perform exercises regardless of the symptoms (time-contingent approach). A detailed description of this treatment approach (i.e., treatment protocol) is published elsewhere [23].

#### 2.7.2. Control Group Intervention Including Biomedically-Focused Treatment

The control intervention combined traditional back/neck school with general physical exercise therapy. Unlike pain neuroscience education that emphasized central pain processing mechanisms, the education in the control group covered mechanical causes of NP and LBP, spine anatomy, physiology, and biomechanics (e.g., the importance of self-care and ergonomics; intra-discal pressure and joint forces; lifting techniques; and the value of stretching), and the importance of strength, endurance, fitness training, the postural strain associated with various postures and daily activities (including standing, sitting, and lifting) [41,42].

After the education, the exercise program focused on possible biomedical dysfunctions of the spine (e.g., mobility, muscle strength, muscle endurance, and general fitness exercises), with a progression toward functional activities and physically demanding tasks. The focus was on keeping the spine in physiologically neutral positions during exercise [41,42]. Importantly—different from the experimental intervention—when symptoms occurred during or after an exercise, the intensity or frequency of the exercise was reduced (pain-contingent approach). For more details on the treatment protocols, we refer to Dolphens et al. (2014) [27] and Malfliet et al. (2017) [23].

### 2.8. Statistical Analysis

All the statistical analyses were performed using SPSS Software (Version 26). Using the Kolmogorov–Smirnov test, data normality was checked. A multivariate analysis of covariance (MANCOVA) was performed to control the effects of handedness and age as possible confounding covariates.

To assess the difference between groups in response to treatment, a random-intercept linear mixed model analysis, using Bonferroni post hoc analyses, was applied with an unstructured covariance matrix. The model included treatment, time, and treatment × time as fixed effects together with a random intercept for each patient. Age was included as a covariate in the model because age has a significant impact on WM structure [43]. *p* < 0.05 (2-sided) was considered significant. Additionally, for each variable, the percentage of change compared with baseline was calculated. Effect sizes of the mean group differences were calculated as the Cohen d.

To analyze the correlations between outcome variables, the differences between WM structural changes (post-intervention minus baseline and follow-up minus baseline) and clinical outcomes improvements (post-intervention minus baseline and follow-up minus baseline) were calculated. Then, using partial correlation analyses with age and treatment as covariates, associations were examined only between WM structural changes on the one hand, and clinical outcomes improvement on the other hand from baseline to post-treatment and baseline to 1-year follow-up (*p* < 0.05). A Bonferroni correction approach was used for multiple comparisons by the following formula: (*α-corr* = 0.05/k) where k = the number of comparisons on the same dependent outcome.

## 3. Results

### 3.1. Study Population

**Figure 2:**
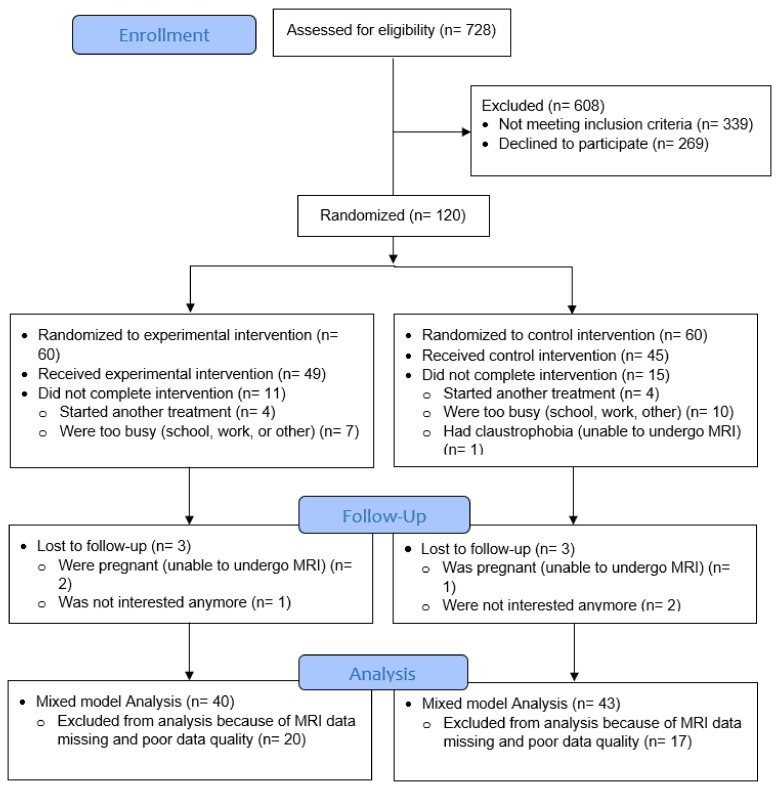
CONSORT flow diagram of this study.

Out of 120 participants, 32 cases were lost-to-follow-up post-treatment and/or at 1-year post-baseline. Poor data quality was observed in 24 cases because of too much head translation due to head motion (exceeding the size of 1 voxel) and general low data quality profile (spikes). If the baseline assessment was of poor quality, the follow-up data of that specific participant was also excluded from the dataset. In case of poor data quality at 1-year follow-up, other assessments of that participant were included in the analysis using linear mixed models to correct for the data loss. Hence, the final number of participants for linear mixed model analysis entailed n = 40 for the experimental group and n = 43 for the control group (Figure 2).

For partial correlation analysis, because MRI data were missing, the number of participants decreased to 54 (27 in each group) from baseline to post-treatment and decreased to 50 (26 for the experimental group and 24 for the control group) from baseline to 1-year follow-up.

The MANCOVA with age and handedness as covariates revealed no significant main effect of handedness on WM structure (*p =* 0.817 for FA; *p =* 0.526 for MD; *p =* 0.701 for AD; *p =* 0.697 for RD). Therefore, WM structure data of the left (n = 3) and right-handed (n = 80) study participants were analyzed together.

The results of the effects of the interventions on clinical outcomes and the reasons for lost-to-follow-up of the present RCT have been published elsewhere [22]. The previous analyses showed that the MPNA was an effective treatment to reduce pain, symptoms related to central sensitization, disability, and kinesiophobia, and increase function and PPTs with small to medium effect sizes.

Group demographics were compared using an independent sample *t*-test or chi-squared test and no significant differences were observed between groups (Table 1).

### 3.2. Time and Treatment Effects on the Values of WM ROIs from Baseline to Post-Treatment and Baseline to 1-Year Follow-Up

Because there were significant effects of age on many of the diffusion measures, age was entered into the linear mix model as a covariate. All statistics reported below were generated after controlling for age and are presented with details in Table S1, in Supplementary Materials.

The linear mixed model analysis showed no significant main effect of treatment and no significant interaction effects (all *p*-values > 0.05). However, for various WM ROIs, a significant main effect of time was found, and Bonferroni post-hoc comparisons were carried out to identify the exact change (Table 2).

A decrease from baseline to 1-year follow-up in FA was seen for both treatment groups in the anterior corona radiata in the left (F = 13.20, *p <* 0.001) and right (F = 18.55, *p <* 0.001) hemispheres, while an increase from baseline to 1-year follow-up in FA was found in the cingulum hippocampus in the left hemisphere (F = 3.73, *p =* 0.03).

For MD, an increase from baseline to 1-year follow-up was found for both groups in the anterior corona radiata in the left (F = 13.99, *p <* 0.001) and right (F = 29.53, *p <* 0.001) hemispheres, the anterior limb of internal capsule in the right hemisphere (F = 6.22, *p <* 0.001), and the superior cerebellar peduncle in the left hemisphere (F = 3.38, *p =* 0.04). Additionally, a decrease in MD from baseline to 1-year follow-up was found for both groups in the cingulum hippocampus in the left hemisphere (F = 4.20, *p* = 0.02). One ROI that showed a significant main effect of time for MD, which disappeared after Bonferroni post hoc tests, was the retrolenticular part of the internal capsule in the right hemisphere.

In terms of AD, an increase from baseline to 1-year follow-up was seen for the anterior corona radiata in the right (F = 15.70, *p <* 0.001) hemisphere, the retrolenticular part of the internal capsule in the right hemisphere (F = 5.45, *p <* 0.001), and the superior longitudinal fasciculus in the right hemisphere (F = 18.28, *p <* 0.001). The significant main effect of time found for the anterior corona radiate in the left hemisphere and the anterior limb of the internal capsule in the right hemisphere disappeared after Bonferroni post hoc tests.

Lastly, RD values increased from baseline to 1-year follow-up in the anterior corona radiata in the left (F = 17.44, *p <* 0.001) and right (F = 29.61, *p <* 0.001) hemispheres and the cingulum (cingulate gyrus) in the right hemisphere (F = 3.60, *p =* 0.03), while they decreased from baseline to 1-year follow-up in the cingulum hippocampus (F = 4.23, *p =* 0.01) and superior cerebellar peduncle in the left hemisphere (F = 3.46, *p =* 0.03).

### 3.3. Correlation Analysis Between the Change in Values of WM ROIs and Changes in Clinical Outcomes from Baseline to 1-Year Follow-Up Across Both Groups

To limit the number of analyses performed, correlation analyses were only carried out using WM outcomes that showed a significant effect in the linear mixed model analyses (Table 2). Moreover, as we only detected changes in WM structures from baseline to 1-year follow-up, we performed the partial correlation analyses (across both groups with age and treatment as covariates) only in this period of time.

**Figure 3:**
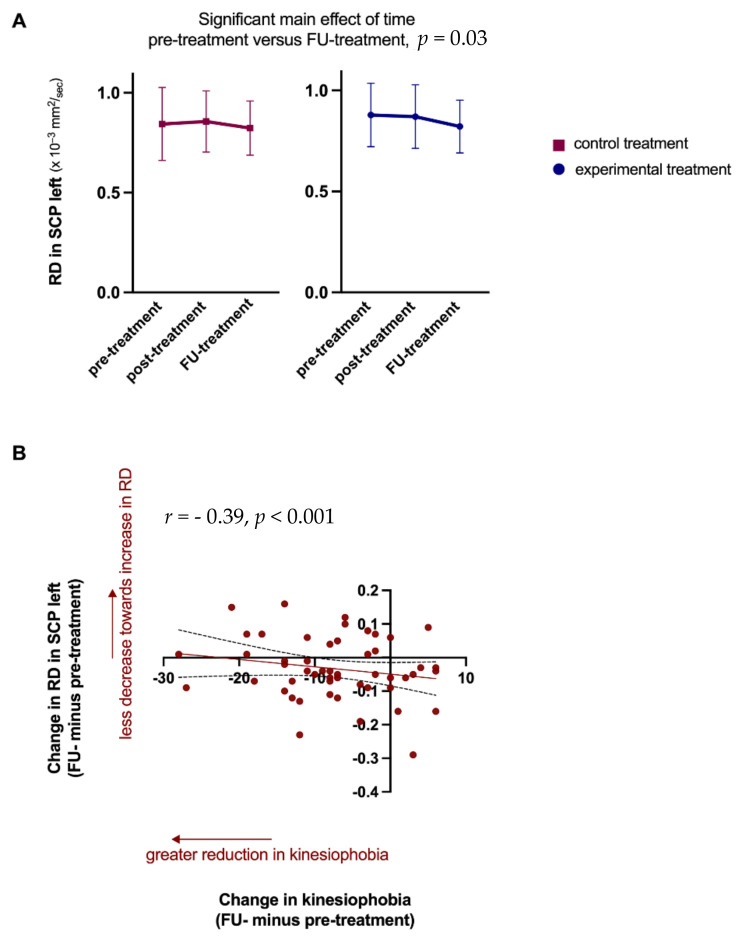
Significant correlation between the change in white matter (WM) structure and change in kinesiophobia from baseline to 1-year follow-up (n = 50). The WM structural change after the control and experimental treatment in which changes over time were significantly correlated with change in kinesiophobia is shown; mean and standard deviation (SD) are presented (**A**). The change in RD values in SCP left are plotted against changes in kinesiophobia to visualize the association (**B**). The significant correlation survives Bonferroni correction for multiple comparisons (alpha = 0.05/19 = 0.0026). SCP = superior cerebellar peduncle, RD = radial diffusivity, FU = follow-up.

Results of the differences between WM structural changes and clinical outcomes’ improvements are presented in Table 3. Partial correlation analysis from baseline to 1-year follow-up showed a significant negative moderate correlation between RD changes of the superior cerebellar peduncle in the left hemisphere with TSK scores (r = −0.39, *p* < 0.001) (Figure 3 and Table 4).

## 4. Discussion

This study investigated whether the MPNA, compared to biomedically-focused treatment, can change WM structure in people suffering from nonspecific CSP. No significant interaction effects were found. Looking into the significant main effect of time, the main changes were revealed in the anterior corona radiata, the anterior limb of the internal capsule, and the retrolenticular part of the internal capsule, where a decrease in FA value and an increase in MD and AD values were found from baseline to 1-year follow-up. Notably, only in the cingulum hippocampus was an increase in FA value together with a decrease in MD and RD value observed from baseline to 1-year follow-up.

Interestingly, Mansour et al. (2013) showed lower FA and higher MD and RD in various WM structures including the anterior corona radiata, the anterior limb of the internal capsule, the retrolenticular part of the internal capsule, and the superior longitudinal fasciculus regions in patients with LBP whose pain became persistent [19]. Therefore, the changes that we found in WM properties over time in these regions could probably be better explained by the chronic pain diagnosis combined with the progression of time, and not by a specific treatment effect. It is also possible that CSP affects WM tracts that were not selected as ROIs. For example, a recent study in patients with CLBP found decreased WM FA in the corpus callosum adjacent to the anterior cingulate in the virtual reality neuroscience-based therapy group, relative to the control condition [44].

Additionally, the fact that we could only find one significant correlation between the changes in WM properties and the clinical improvements in response to treatment questions the clinical importance of the observed WM changes, similar to our previous findings showing no detectable changes in gray matter morphologic features in this population in response to treatment [22]. The direction of the significant association is interesting because a greater reduction in kinesiophobia was correlated with a smaller decrease toward an increase in RD in the left superior cerebellar peduncle in patients with CSP. This WM tract showed across both treatment groups a decrease in RD at 1-year follow-up compared to baseline. It could also be that other WM regions/tracts or using more advanced diffusion-weighted imaging techniques could have shown other results or that the findings suggest limitations in using DTI for monitoring treatment effects in CSP.

### 4.1. Changes in the Values of WM ROIs over Time in Patients with CSP

The primary diffusion metrics (FA and MD) are suggested to reflect overall WM health, organization, and maturation [45]. AD reflects axon integrity while RD reflects myelin sheath integrity and both have important roles in understanding the underlying physiological brain mechanisms [46]. Lower FA demonstrates lower microstructural integrity of the WM pathways, and WM neurodegenerative pathology is frequently associated with lower FA within specific tracts [47]. In addition, the associations of lower FA and higher MD and RD with neuronal plasticity and cortical reorganization have been observed in chronic pain conditions such as complex regional pain syndrome [15,48]. Neuronal plasticity and cortical reorganization are also observed in patients with CSP [49,50] that result in maladaptive processing of sensory information in the central nervous system and have implications for a decreased sensory acuity [49]. Moreover, lower FA has been observed to be associated with the risk of pain chronification in subacute LBP patients [19]. Considering the relation of WM structural changes and its effect on impaired sensory acuity and state of pain chronification, it seems that DTI metrics changed by the pain chronification diagnosis in time [19] and not by the specific treatment effect.

Our results showed that the main regions with WM structural changes (a decrease in FA and an increase in MD, AD, and RD) were the anterior corona radiata, the anterior limb of the internal capsule, and the retrolenticular part of the internal capsule, which are parts of the internal capsule. The internal capsule is a WM structure located in the inferomedial portion of each cerebral hemisphere and is composed of both motor and sensory fibers that connect the thalamus and brain stem with the cerebral and prefrontal cortex [51]. Fiber tracts in the internal capsule are associated with the processing of emotions, cognition, decision-making, and motivation, and, based on DTI studies, changes in WM structure in this area are also associated with the occurrence of depression and functional impairments [52,53]. On the other hand, evidence has indicated an association between depression, abnormalities in cognitive functions, and decision-making in patients with CLBP [54,55,56]. Therefore, it can be speculated that, in the absence of an effective treatment to change WM structure, the main changes in WM structure have been found in the internal capsule region with the progression of time.

The cingulum hippocampus was the only region with different results (an increase in FA value and a decrease in MD, AD, and RD values, whereas the other regions showed a decrease in FA and an increase in the other WM properties). The cingulum is a prominent WM tract that supports prefrontal, parietal, and temporal lobe interactions. The cingulum includes motor and sensory fibers associated with the cingulate cortices that connect with the thalamus, dorsolateral prefrontal cortex, insula, parahippocampal cortices, inferior component of the hippocampal formation, and amygdala. Consistent with our findings, Kennis et al. (2015) investigated trauma-focused therapy effects on the WM structure of the cingulum bundle and found treatment-by-time interaction effects on increasing FA values in hippocampal cingulum in persistent posttraumatic stress disorder patients [57]. However, to compare the current study’s results, there is a lack of studies investigating the effect of treatment on WM structure in this region in patients with chronic pain.

### 4.2. Association of Changes in the Values of WM ROIs with Improvements in Clinical Outcomes

Changes in RD value in the left superior cerebellar peduncle showed a negative association with the magnitude of improvement in kinesiophobia after 1-year follow-up. Previous studies reported varied results. Ceko et al. (2015) found that increased FA values in the insula post-treatment (spine surgery or facet joint injections) versus pre-treatment were associated with more pain improvement in patients with CLBP [58]. Lewis et al. (2018) found, in their longitudinal study, no significant association between FA values across the whole-brain WM with clinical outcomes in patients with knee osteoarthritis after knee total arthroplasty [59]. However, studies investigating associations between changes in WM structure and changes in clinical outcomes following multimodal conservative interventions for chronic pain are scarce; hence, more research is required.

### 4.3. Strengths and Limitations

There are several strengths to our study. First, this is the first study to investigate whether an intervention that focused on central pain processing was effective for improving clinical outcomes and can also be effective in changing WM structure, compared to biomedically-focused treatment, in patients with nonspecific CSP. Second, the generalizability of our results is enhanced because participants were randomly allocated to the interventions, conducted in multiple primary care centers.

However, the current study also has some limitations that need to be addressed. First, although the DTI technique provides new information on the fiber tract structure, it has some technical limitations due to the “crossing and termination” problems [60]. DTI cannot adequately characterize diffusion in regions of complex fiber architecture such as crossing fibers [60]. In addition, single-shell DWI data reconstructed with the diffusion tensor model were acquired which underestimates diffusion restriction in voxels within crossing fibers and makes the interpretation of the changes on a cellular scale very challenging. For example, it remains impossible with single-shell DTI to differentiate myelination from compaction or an increase in the number of fibers, which would all result in an increase in FA [61]. These limitations can be addressed by some emerging fiber mapping techniques, such as multi-shell and diffusion spectrum imaging, which provide more information about the underlying tissue microstructure [62].

Second, having no pain-free control group in this study, it was not possible to assess whether there were structural WM differences between the included patients with CSP and healthy controls. Also, including a pain-free control group receiving no treatment, and scanning this group at baseline and at time points matched with the assessment time points of the patients, could yield more insight into the effect of time on the WM structural changes.

Third, this study lacked a 1-year follow-up measurement of PPTs (i.e., pressure pain sensitivity) that could have provided insight into possible associations between WM structural changes and changes in pressure pain sensitivity.

### 4.4. Further Research

Further research using Neurite Orientation Dispersion and Density Imaging and multi-shell high angular resolution diffusion imaging (HARDI), allowing for a more specific examination of WM microstructural changes after multi-modal treatment approaches, combining cognitive–behavioral and physical therapy in patients with chronic pain, would be relevant.

## 5. Conclusions

In conclusion, although the previously reported RCT showed that the MPNA was more effective than biomedically-focused treatment for improving clinical outcomes in patients with CSP, significant changes in WM structure in various ROIs were observed over time, irrespective of the treatment groups. Only one significant moderate correlation was found between WM structural change in the superior cerebellar peduncle and changes in kinesiophobia. Overall, our findings can question the relevance of WM structural changes during the improvement of clinical outcomes in patients with CSP.

## Figures and Tables

**Table 1:** Demographic and baseline data of patients with nonspecific chronic spinal pain included in the analyses.

Data	Control Group (n = 43)	Experimental Group (n = 40)	*p* Value
Age (y) *	38.91 ± 11.92	39.32 ± 11.30	0.96
Weight (kg) *	71.72 ± 14.61	68.72 ± 14.51	0.72
Height (cm) *	172.53 ± 10.91	172.03 ± 9.06	0.09
Pain duration (month) *	111.21 ± 84.82	124.70 ± 104.03	0.14
Sex No. (%)			0.84
Female	26(60)	28(70)	
Male	17(40)	12(30)	
Dominant pain problem No. (%)			0.16
Neck pain	23(54)	20(50)	
Low back pain	20(46)	20(50)	
Educational level No. (%)			0.67
No education	0	0	
Lower secondary education	6(14)	3(8)	
Higher secondary education	9(21)	5(12)	
Higher education	28(65)	32(80)	
Handedness No. (%)			0.18
Right hand	42(98)	38(95)	
Left hand	1(2)	2(5)	

Significant *p*-value (<0.05). * Value stands for mean ± standard deviation. No. = number.

**Table 2:** Time-effect significant results in FA, MD, AD, and RD values in WM regions of interest in left and right hemispheres.

DTI-Derived Metric (Hemisphere’s Side)		Control Group		Experimental Group		Mean Group Difference (95%CI)	Main Effect of Time	Interaction Effect	Bonferroni Post-Hoc Test
		Mean ± SD	% of Changes Rel. to Baseline	Mean ± SD	% of Changes Rel. to Baseline				
Anterior corona radiate									
FA (LH)	Baseline	0.35 ± 0.02	-	0.35 ± 0.02	-	−0.08 (−0.57, 0.41)	F = 13.20 *p <* 0.001 *	F = 1.04 *p* = 0.35	Baseline to Post-treatment: *p* = 0.13 Baseline to Follow-up: *p <* 0.001 *
	Post-treatment	0.35 ± 0.01	−0.84%	0.35 ± 0.02	1.40%	0 (−0.49, 0.49)			
	Follow-up	0.34 ± 0.02	−1.41%	0.34 ± 0.02	−3.09%	0.17 (−0.32, 0.67)			
FA (RH)	Baseline	0.36 ± 0.01	-	0.36 ± 0.03	-	−0.03 (−0.53, 0.45)	F = 18.55 *p <* 0.001 *	F = 0.38 *p* = 0.68	Baseline to Post-treatment: *p =* 1.00 Baseline to Follow-up: *p <* 0.001 *
	Post-treatment	0.36 ± 0.01	−0.27%	0.359 ± 0.02	−0.82%	0.03 (−0.46, 0.53)			
	Follow-up	0.35 ± 0.01	1.10%	0.35 ± 0.02	−3.03%	0.25 (−0.24, 0.75)			
MD (LH)	Baseline	0.73 ± 0.01	-	0.73 ± 0.02	-	−0.16 (−0.66, 0.33)	F = 13.99 *p <* 0.001 *	F = 1.12 *p* = 0.33	Baseline to Post-treatment: *p =* 1.00 Baseline to Follow-up: *p <* 0.001 *
	Post-treatment	0.73 ± 0.02	0.41%	0.73 ± 0.03	0.13%	−0.07 (−0.57, 0.42)			
	Follow-up	0.73 ± 0.02	1.09%	0.74 ± 0.02	1.36%	−0.24 (−0.74, 0.25)			
MD (RH)	Baseline	0.72 ± 0.02	-	0.73 ± 0.02	-	−0.17 (−0.67, 0.32)	F = 29.53 *p <* 0.001 *	F = 1.14 *p* = 0.24	Baseline to Post-treatment: *p =* 1.00 Baseline to Follow-up: *p <* 0.001 *
	Post-treatment	0.72 ± 0.02	0.27%	0.72 ± 0.02	−0.41%	−0.03 (−0.53, 0.45)			
	Follow-up	0.73 ± 0.02	1.51%	0.74 ± 0.02	1.78%	−0.25 (−0.75, 0.24)			
AD (LH)	Baseline	1.01 ± 0.02	-	1.02 ± 0.02	-	−0.43 (−0.93, 0.07)	F = 4.30 *p <* 0.001 *	F = 0.32 *p* = 0.72	Baseline to Post-treatment: *p* = 0.12 Baseline to Follow-up: *p* = 1.000
	Post-treatment	1.01 ± 0.02	0%	1.02 ± 0.02	.19%	−0.52 (−0.52, −0.01)			
	Follow-up	1.02 ± 0.02	1.08%	1.02 ± 0.01	0%	0.04 (−0.45, 0.54)			
AD (RH)	Baseline	1.02 ± 0.02	-	1.02 ± 0.02	-	0.07 (−0.42, 0.57)	F = 15.70 *p <* 0.001 *	F = 1.61 *p* = 0.20	Baseline to Post-treatment: *p* = 0.97 Baseline to Follow-up: *p* = 0.001 *
	Post-treatment	1.02 ± 0.02	−0.29%	1.02 ± 0.02	0.29%	−0.15 (−0.64, 0.34)			
	Follow-up	1.03 ± 0.02	0.97%	1.03 ± 0.02	1.36%	−0.09 (−0.58, 0.40)			
RD (LH)	Baseline	0.58 ± 0.02	-	0.58 ± 0.03	-	−0.06 (−0.56, 0.42)	F = 17.44 *p <* 0.001 *	F = 1.40 *p* = 0.22	Baseline to Post-treatment: *p* = 1.00 Baseline to Follow-up: *p <* 0.001 *
	Post-treatment	0.59 ± 0.02	0.68%	0.59 ± 0.03	0.68%	−0.06 (−0.56, 0.43)			
	Follow-up	0.59 ± 0.02	1.87%	0.60 ± 0.03	2.21%	−0.13 (−0.632 0.36)			
RD (RH)	Baseline	0.58 ± 0.02	-	0.58 ± 0.03	-	0.21 (−0.28, 0.71)	F = 29.61 *p <* 0.001 *	F = 0.88 *p* = 0.42	Baseline to Post-treatment: *p* = 1.00 Baseline to Follow-up: *p <* 0.001 *
	Post-treatment	0.58 ± 0.02	−1.19%	0.58 ± 0.03	−0.17%	0 (−0.49, −0.48)			
	Follow-up	0.58 ± 0.02	0.34%	0.59 ± 0.03	2.75%	−0.26 (−0.76, 0.23)			
Anterior limb of internal capsule									
MD (RH)	Baseline	0.68 ± 0.01	-	0.68 ± 0.01	-	0 (−0.49, 0.49)	F = 6.22 *p* < 0.001 *	F = 1.47 *p* = 0.23	Baseline to Post-treatment: *p* = 1.00 Baseline to Follow-up: *p* = 0.03 *
	Post-treatment	0.69 ± 0.02	0.29%	0.68 ± 0.01	−0.43%	0.23 (−0.26, 0.73)			
	Follow-up	0.69 ± 0.02	0.72%	0.69 ± 0.02	0.87%	−0.04 (−0.54, 0.45)			
AD (RH)	Baseline	1.07 ± 0.03	-	1.08 ± 0.03	-	−0.34 (−0.84, 0.15)	F = 4.41 *p* = 0.01 *	F = 3.09 *p* = 0.054	Baseline to Post-treatment: *p* = 1.00 Baseline to Follow-up: *p* = 0.12
	Post-treatment	1.08 ± 0.03	0.65%	1.08 ± 0.03	−0.27%	−0.02 (−0.52, 0.47)			
	Follow-up	1.08 ± 0.03	1.21%	1.09 ± 0.03	.55%	−0.11 (−0.61, 0.38)			
Cingulum (cingulate gyrus)									
RD (RH)	Baseline	0.60 ± 0.04	-	0.60 ± 0.04	-	0.12 (−0.37, 0.61)	F = 3.60 *p* = 0.03 *	F = 1.02 *p* = 0.36	Baseline to Post-treatment: *p* = 1.00 Baseline to Follow-up: *p* = 0.04 *
	Post-treatment	0.61 ± 0.04	1.48%	0.60 ± 0.03	0.33%	0.29 (−0.20, 0.79)			
	Follow-up	0.61 ± 0.04	1.98%	0.61 ± 0.03	1.83%	0.14 (−0.28, 0.57)			
Cingulum hippocampus									
FA (LH)	Baseline	0.24 ± 0.03	-	0.24 ± 0.03	-	0 (−0.42, 0.42)	F = 3.73 *p* = 0.03 *	F = 2.13 *p* = 0.12	Baseline to Post-treatment: *p* = 1.00 Baseline to Follow-up: *p* = 0.04 *
	Post-treatment	0.24 ± 0.03	0%	0.25 ± 0.02	2.02%	−0.17 (−0.60, 0.24)			
	Follow-up	0.26 ± 0.02	5.26%	0.25 ± 0.02	1.61%	0.34 (−0.08, 0.77)			
MD (LH)	Baseline	0.84 ± 0.08	-	0.85 ± 0.06	-	−0.06 (−0.49, 0.35)	F = 4.20 *p* = 0.02 *	F = 1.51 *p* = 0.22	Baseline to Post-treatment: *p* = 0.99 Baseline to Follow-up: *p* = 0.01 *
	Post-treatment	0.84 ± 0.09	−0.82%	0.85 ± 0.05	−0.35%	−0.11 (−0.54, 0.30)			
	Follow-up	0.81 ± 0.05	−3.77%	0.84 ± 0.06	−0.46%	−0.54 (−0.97, −0.10)			
RD (LH)	Baseline	0.74 ± 0.08	-	0.74 ± 0.07	-	−0.08 (−0.51, 0.33)	F = 4.23 *p* = 0.01 *	F = 0.88 *p* = 0.41	Baseline to Post-treatment: *p* = 1.00 Baseline to Follow-up: *p* = 0.01 *
	Post-treatment	0.73 ± 0.09	−0.94%	0.74 ± 0.06	−1.06%	0.07 (−0.35, 0.50)			
	Follow-up	0.71 ± 0.06	−4.31%	0.73 ± 0.07	−1.73%	−0.36 (−0.79, 0.06)			
Retrolenticular part of the internal capsule									
MD (RH)	Baseline	0.71 ± 0.04	-	0.71 ± 0.02	-	0.08 (−0.34, 0.51)	F = 3.62 *p* = 0.03 *	F = 0.17 *p* = 0.84	Baseline to Post-treatment: *p* = 1.00 Baseline to Follow-up: *p* = 0.20
	Post-treatment	0.71 ± 0.03	−0.27%	0.71 ± 0.02	−0.28%	0.10 (−0.32, 0.53)			
	Follow-up	0.71 ± 0.02	0.13%	0.71 ± 0.02	0.84%	−0.09 (−0.51, 0.33)			
AD (RH)	Baseline	1.12 ± 0.05	-	1.11 ± 0.03	-	0.25 (−0.17, 0.68)	F = 5.45 *p* = 0.007 *	F = 0.94 *p* = 0.39	Baseline to Post-treatment: *p* = 1.00 Baseline to Follow-up: *p* = 0.03 *
	Post-treatment	1.12 ± 0.05	−0.53%	1.12 ± 0.04	0.53%	−0.02 (−0.44, 0.40)			
	Follow-up	1.13 ± 0.04	.35%	1.12 ± 0.03	1.07%	0.07 (−0.35, 0.50)			
Superior cerebellar peduncle									
MD (LH)	Baseline	1.06 ± 0.15	-	1.08 ± 0.15	-	−0.13 (−0.56, 0.29)	F = 3.38 *p* = 0.04 *	F= 1.10 *p* = 0.33	Baseline to Post-treatment: *p* = 1.00 Baseline to Follow-up: *p* = 0.04 *
	Post-treatment	1.08 ± 0.14	1.87%	1.10 ± 0.14	1.65%	−0.128 (−0.55, 0.90)			
	Follow-up	1.05 ± 0.13	0.65%	1.04 ± 0.11	−3.31%	0.06 (−0.36, 0.49)			
RD (LH)	Baseline	0.83 ± 0.16	-	0.86 ± 0.15	-	−0.18 (−0.61, 0.24)	F = 3.46 *p* = 0.03 *	F = 1.12 *p* = 0.33	Baseline to Post-treatment: *p* = 0.73 Baseline to Follow-up: *p* = 0.03 *
	Post-treatment	0.85 ± 0.15	2.76%	0.87 ± 0.15	0.92%	−0.09 (−0.52, 0.33)			
	Follow-up	0.82 ± 0.13	−1.20%	0.82 ± 0.13	−4.86%	0.01 (−0.41, 0.44)			
Superior longitudinal fasciculus									
AD (RH)	Baseline	0.98 ± 0.02	-	0.98 ± 0.02	-	0.08 (−0.34, 0.51)	F = 18.28 *p* < 0.001 *	F = 1.97 *p* = 0.15	Baseline to Post-treatment: *p* = 1.00 Baseline to Follow-up: *p <* 0.001 *
	Post-treatment	0.98 ± 0.02	0%	0.98 ± 0.02	−0.2%	0.14 (−0.28, 0.57)			
	Follow-up	0.99 ± 0.02	1.21%	0.99 ± 0.02	1.22%	0.07 (−0.35, 0.50)			

All analyses were performed using Linear Mixed Models. When a variable was assessed more than two times ‘repeated covariance type’ was set at ‘unstructured’. Age was entered as a covariate. Significant *p*-values are visualized with *. MD, AD, and RD are in units of 10^−3^ mm^2^/s. Abbreviations: WM = white matter, DTI = diffusion tensor imaging FA = fractional anisotropy, MD = mean diffusivity, AD = axial diffusivity, RD = radial diffusivity, LH = left hemisphere, RH = right hemisphere. Follow-up = 12 months after the start of treatment.

**Table 3:** Results of the differences of WM structural changes and clinical outcomes change from baseline to post-treatment and baseline to 1-year follow-up.

Clinical Outcomes	Post-Treatment Minus Baseline	Follow-Up Minus Baseline
	Mean ± SD	Mean ± SD
NRS (/10)	−2.41 ± 2.16	−2.20 ± 2.31
CSI (/100)	−7.95 ± 9.43	−9.12 ± 12.00
PDI (/70)	−11.69 ± 11.82	−12.91 ± 12.51
SFPH (/400)	52.77 ± 88.47	56.80 ± 93.70
SFMH (/400)	10.80 ± 92.90	9.68 ± 106.49
PCS (/52)	−6.17 ± 8.10	−8.54 ± 9.96
TSK (/68)	−7.87 ± 8.54	−8.56 ± 9.48
PPT upper trapezius (kgf)	0.37 ± 2.38	-
PPT lumbar spine (kgf)	0.45 ± 2.92	-
PPT quadriceps (kgf)	0.09 ± 2.56	-
**DTI-derived metric (hemisphere’s side)**		
FA in Anterior corona radiate (LH)	−0.003 ± 0.012	−0.01 ± 0.01
FA in Anterior corona radiate (RH)	−0.005 ± 0.01	−0.004 ± 0.01
FA in Cingulum hippocampus (LH)	0.002 ± 0.02	−0.03 ± 0.11
MD in Anterior corona radiate (LH)	−0.002 ± 0.01	0.003 ± 0.01
MD in Anterior corona radiate (RH)	0.001 ± 0.01	0.01 ± 0.01
MD in Anterior limb of internal capsule (RH)	−0.001 ± 0.01	0.01 ± 0.05
MD in Cingulum hippocampus (LH)	−0.004 ± 0.04	−0.01 ± 0.04
MD in Retrolenticular part of internal capsule (RH)	−0.001 ± 0.02	0.007 ± 0.02
MD in Superior cerebellar peduncle (LH)	−0.01 ± 0.08	−0.03 ± 0.09
AD in Anterior corona radiate (LH)	−0.004 ± 0.01	0.00 ± 0.01
AD in Anterior corona radiate (RH)	−0.003 ± 0.01	−0.01 ± 0.14
AD in Anterior limb of internal capsule (RH)	−0.00 ± 0.02	0.003 ± 0.02
AD in Retrolenticular part of internal capsule (RH)	−0.001 ± 0.03	0.007 ± 0.02
AD in Superior longitudinal fasciculus (RH)	−0.002 ± 0.01	0.01 ± 0.01
RD in Anterior corona radiate (LH)	0.001 ± 0.01	0.01 ± 0.01
RD in Anterior corona radiate (RH)	−0.00 ± 0.01	0.01 ± 0.01
RD in Cingulum cingulate gyrus (RH)	0.003 ± 0.04	0.004 ± 0.04
RD in Cingulum hippocampus (LH)	−0.005 ± 0.04	−0.01 ± 0.04
RD in Superior cerebellar peduncle (LH)	−0.01 ± 0.08	−0.02 ± 0.08

MD, AD, and RD are in units of 10^−3^ mm^2^/s. Experimental and control were grouped together. Abbreviations: SD = standard deviation, NRS = numeric rating scale, CSI = central sensitization inventory, PDI = pain disability index, SFPH = short form physical health survey, SFMH = short form mental health survey, PCS = pain catastrophizing scale, TSK = tampa scale for kinesiophobia, PPT = pressure pain thresholds, WM = white matter, FA = fractional anisotropy, MD = mean diffusivity, AD = axial diffusivity, RD = radial diffusivity, LH = left hemisphere, and RH = right hemisphere.

**Table 4:** Partial correlations of significant FA, MD, AD, and RD values in WM regions with clinical outcomes from baseline to 1-year follow-up across both treatment groups.

DTI-Derived Metric (Hemisphere’s Side)	NRS	CSI	PDI	SFPH	SFMH	PCS	TSK
FA in Anterior corona radiata (LH)	−0.36 (*p* = 0.01)	−0.17 (*p* = 0.24)	0.08 (*p* = 0.57)	0.13 (*p* = 0.35)	0.03 (*p* = 0.82)	0.10 (*p* = 0.46)	−0.01 (*p* = 0.94)
FA in Anterior corona radiate (RH)	−0.22 (*p* = 0.13)	0.01 (*p* = 0.90)	0.10 (*p* = 0.50)	0.09 (*p* = 0.53)	−0.05 (*p* = 0.71)	0.09 (*p* = 0.53)	−0.04 (*p* = 0.49)
FA in Cingulum hippocampus (LH)	0.12 (*p* = 0.40)	−0.08 (*p* = 0.57)	−0.05 (*p* = 0.70)	0.05 (*p* = 0.71)	−0.07 (*p =* 60)	−0.05 (*p* = 0.70)	−0.10 (*p* = 0.47)
MD in Anterior corona radiate (LH)	0.02 (*p* = 0.86)	0.01 (*p* = 0.92)	0.02 (*p* = 0.86)	−0.07 (*p* = 0.64)	0.08 (*p* = 0.59)	−0.22 (*p* = 0.13)	0.14 (*p* = 0.34)
MD in Anterior corona radiate (RH)	0.00 (*p* = 0.96)	−0.25 (*p* = 0.08)	−0.09 (*p* = 0.54)	0.07 (*p* = 0.62)	0.06 (*p* = 0.68)	−0.23 (*p* = 0.11)	−0.00 (*p* = 0.99)
MD in Anterior limb of internal capsule (RH)	0.03 (*p* = 0.84)	−0.07 (*p* = 0.62)	0.08 (*p* = 0.56)	0.05 (*p* = 0.70)	−0.04 (*p* = 0.74)	−0.18 (*p* = 0.21)	0.06 (*p* = 0.65)
MD in Cingulum hippocampus (LH)	−0.02 (*p* = 0.88)	−0.06 (*p* = 0.65)	0.16 (*p* = 0.26)	−0.12 (*p* = 0.40)	−0.07 (*p* = 0.61)	0.28 (*p* = 0.05)	0.10 (*p* = 0.47)
MD in Retrolenticular part of internal capsule (RH)	0.08 (*p* = 0.56)	−0.00 (*p* = 0.96)	0.12 (*p* = 0.41)	−0.08 (*p* = 0.57)	0.08 (*p* = 0.57)	0.06 (*p* = 0.65)	0.13 (*p* = 0.36)
MD in Superior cerebellar peduncle (LH)	−0.15 (*p* = 0.58)	0.02 (*p* = 0.31)	0.06 (*p* = 0.88)	0.02 (*p* = 0.67)	0.05 (*p* = 0.84)	0.07 (*p* = 0.74)	−0.32 (*p* = 0.27)
AD in Anterior corona radiate (LH)	−0.28 (*p* = 0.05)	−0.12 (*p* = 0.41)	0.12 (*p* = 0.42)	0.07 (*p* = 0.62)	0.03 (*p* = 0.81)	−0.07 (*p* = 0.62)	0.08 (*p* = 0.58)
AD in Anterior corona radiate (RH)	−0.03 (*p* = 0.79)	−0.19 (*p* = 0.17)	−0.18 (*p* = 0.20)	0.15 (*p* = 0.29)	0.01 (*p* = 0.90)	−0.19 (*p* = 0.19)	0.01 (*p* = 0.92)
AD in Anterior limb of internal capsule (RH)	0.05 (*p* = 0.84)	0.12 (*p* = 0.84)	0.26 (*p* = 0.84)	−0.09 (*p* = 0.84)	−0.03 (*p* = 0.84)	0.11 (*p* = 0.84)	0.10 (*p* = 0.84)
AD in Retrolenticular part of internal capsule (RH)	0.01 (*p* = 0.91)	−0.13 (*p* = 0.35)	0.04 (*p* = 0.76)	0.01 (*p* = 0.90)	0.09 (*p* = 0.50)	0.03 (*p* = 0.80)	0.17 (*p* = 0.25)
AD in Superior longitudinal fasciculus (RH)	−0.01 (*p* = 0.91)	−0.13 (*p* = 0.37)	0.07 (*p* = 0.62)	−0.09 (*p* = 0.54)	0.05 (*p* = 0.73)	−0.07 (*p* = 0.62)	0.02 (*p* = 0.89)
RD in Anterior corona radiate (LH)	0.19 (*p* = 0.18)	0.13 (*p* = 0.38)	0.06 (*p* = 0.66)	−0.13 (*p* = 0.38)	−0.00 (*p* = 0.97)	−0.15 (*p* = 0.29)	0.10 (*p* = 0.50)
RD in Anterior corona radiate (RH)	0.19 (*p* = 0.19)	−0.07 (*p* = 0.63)	−0.06 (*p* = 0.68)	−0.07 (*p* = 0.64)	−0.05 (*p* = 0.69)	−0.10 (*p* = 0.47)	0.17 (*p* = 0.24)
RD in Cingulum cingulate gyrus (RH)	0.01 (*p* = 0.92)	0.01 (*p* = 0.92)	−0.06 (*p* = 0.64)	−0.15 (*p* = 0.30)	0.21 (*p* = 0.15)	0.06 (*p* = 0.67)	−0.05 (*p* = 0.73)
RD in Cingulum hippocampus (LH)	−0.03 (*p* = 0.84)	−0.11 (*p* = 0.45)	0.09 (*p* = 0.52)	−0.10 (*p* = 0.50)	−0.08 (*p* = 0.58)	0.28 (*p* = 0.05)	0.11 (*p* = 0.45)
RD in Superior cerebellar peduncle (LH)	−0.18 (*p* = 0.20)	−0.02 (*p* = 0.85)	0.03 (*p* = 0.79)	0.06 (*p* = 0.68)	0.08 (*p* = 0.59)	0.06 (*p* = 0.67)	−0.39 (*p <* 0.001 *)

Significant *p*-values are visualized with an *. * Survives Bonferroni correction for multiple comparison (alpha = 0.05/19 = 0.0026). Age and treatment were entered as a covariate. MD, AD, and RD are in units of 10^−3^ mm^2^/s. Values represented are the correlation coefficient (P values). Correlation from 0.0 to 0.30, 0.30 to 0.50, and above 0.50 were respectively considered as small, moderate, and strong. Abbreviations: NRS = numeric rating scale, CSI = central sensitization inventory, PDI = pain disability index, SFPH = short form physical health survey, SFMH = short form mental health survey, PCS = pain catastrophizing scale, TSK = tampa scale for kinesiophobia, WM = white matter, FA = fractional anisotropy, MD = mean diffusivity, AD = axial diffusivity, RD = radial diffusivity, LH = left hemisphere, and RH = right hemisphere.

## Data Availability

The data presented in this study are available upon reasonable request from the corresponding author due to privacy reasons.

## Supplementary Materials

The following supporting information can be downloaded at https://www.mdpi.com/article/10.3390/jcm14030867/s1: Table S1: Linear mix model analysis results of FA, MD, AD, and RD values in WM regions of interest in left and right hemisphere.
