# Supplementary material for: Can Pain Neuroscience Education Combined with Cognition-Targeted Exercise Therapy Change White Matter Structure in People with Chronic Spinal Pain? A Randomized Controlled Trial
Source: J Clin Med. 2025 Jan 28;14(3):867. doi: 10.3390/jcm14030867 (PMC11818553; doi:10.3390/jcm14030867)
Supplement: Supplementary file 1 [file jcm-14-00867-s001.zip › Supplementary tables.pdf]

Table S1. Linear mix model analysis results of FA, MD, AD, and RD values in WM regions of interest in left and right hemisphere

DTI-derived metric (hemisphere's side)		Control group		Experimental group		Mean group difference [95%CI]	Main effect of time	Interaction effect	Bonferroni Post-Hoc test
		**Mean±SD**	**% of Changes rel. to baseline**	**Mean±SD**	**% of Changes rel. to baseline**				
Anterior corona radiate									
**FA (LH)**	Baseline	.35±.02	-	.35±.02	-	-.08 [-.57, .41]	F= 13.20 **p< .001**	F= 1.04 p= .35	Baseline to Post-treatment: p=.13 Baseline to Follow-up: **p< .001**
	Post-treatment	.35±.01	−.84%	.35±.02	1.40%	0 [-.49, .49]			
	Follow-up	.34±.02	−1.41%	.34±.02	−3.09%	.17 [-.32, .67]			
**FA (RH)**	Baseline	.36±.01	-	.36±.03	-	-.03 [-.53, .45]	F= 18.55 **p< .001**	F= .38 p= .68	Baseline to Post-treatment: p=1.00 Baseline to Follow-up: **p< .001**
	Post-treatment	.36±.01	−.27%	.359±.02	−.82%	.03 [-.46, .53]			
	Follow-up	.35±.01	1.10%	.35±.02	−3.03%	.25 [-.24, .75]			
**MD (LH)**	Baseline	.73±.01	-	.73±.02	-	-.16 [-.66, .33]	F= 13.99 **p< .001**	F= 1.12 p= .33	Baseline to Post-treatment: p=1.00 Baseline to Follow-up: **p< .001**
	Post-treatment	.73±.02	.41%	.73±.03	.13%	-.07 [-.57, .42]			
	Follow-up	.73±.02	1.09%	.74±.02	1.36%	-.24 [-.74, .25]			
**MD (RH)**	Baseline	.72±.02	-	.73±.02	-	-.17 [-.67, .32]	F= 29.53 **p< .001**	F= 1.14 p= .24	Baseline to Post-treatment: p=1.00 Baseline to Follow-up: **p< .001**
	Post-treatment	.72±.02	.27%	.72±.02	−.41%	-.03 [-.53, .45]			
	Follow-up	.73±.02	1.51%	.74±.02	1.78%	-.25 [-.75, .24]			
**AD (LH)**	Baseline	1.01±.02	-	1.02±.02	-	-.43 [-.93, .07]	F= 4.30 **p< .001**	F= .32 p= .72	Baseline to Post-treatment: p=.12 Baseline to Follow-up: p=1.000
	Post-treatment	1.01±.02	0%	1.02±.02	.19%	-.52 [-.52, -.01]			
	Follow-up	1.02±.02	1.08%	1.02±.01	0%	.04 [-.45, .54]			

**AD (RH)**	Baseline	1.02±.02	-	1.02±.02	-	.07 [-.42, .57]	F= 15.70 **p< .001**	F= 1.61 p= .20	Baseline to Post-treatment: p=.97 Baseline to Follow-up: **p=.001**
	Post-treatment	1.02±.02	−.29%	1.02±.02	.29%	-.15 [-.64, .34]			
	Follow-up	1.03±.02	.97%	1.03±.02	1.36%	-.09 [-.58, .40]			
**RD (LH)**	Baseline	.58±.02	-	.58±.03	-	-.06 [-.56, .42]	F= 17.44 **p< .001**	F= 1.40 p= .22	Baseline to Post-treatment: p=1.00 Baseline to Follow-up: **p< .001**
	Post-treatment	.59±.02	.68%	.59±.03	.68%	-.06 [-.56, .43]			
	Follow-up	.59±.02	1.87%	.60±.03	2.21%	-.13 [-.632 .36]			
**RD (RH)**	Baseline	.58±.02	-	.58±.03	-	.21 [-.28, .71]	F= 29.61 **p< .001**	F= .88 p= .42	Baseline to Post-treatment: p=1.00 Baseline to Follow-up: **p< .001**
	Post-treatment	.58±.02	−1.19%	.58±.03	−.17%	0 [-.49, -.48]			
	Follow-up	.58±.02	.34%	.59±.03	2.75%	-.26 [-.76, .23]			
Anterior limb of internal capsule									
**FA (LH)**	Baseline	.43±.03	-	.43±.03	-	-.05 [-.55, .43]	F= .25 p= .74	F= 2.30 p= .10	
	Post-treatment	.43±.02	−.91%	.43±.03	.22%	-.23 [-.73, .26]			
	Follow-up	.43±.03	−.45%	.43±.03	.22%	-.14 [-.64, .35]			
**FA (RH)**	Baseline	.45±.03	-	.46±.03	-	-.28 [-.79, .21]	F= .52 p= .59	F= .10 p= .89	
	Post-treatment	.46±.03	.43%	.46±.03	0%	-.24 [-.74, .25]			
	Follow-up	.46±.04	.87%	.46±.03	−1.06%	-.02 [-.52, .47]			
**MD (LH)**	Baseline	.68±.01	-	.68±.02	-	-.10 [-.60, .39]	F= 1.02 p= .36	F= 2.42 p= .99	
	Post-treatment	.68±.01	.43%	.68±.01	−.58%	.26 [-.23, .76]			
	Follow-up	.68±.01	.29%	.69±.02	.43%	-.15 [-.64, .34]			
**MD (RH)**	Baseline	.68±.01	-	.68±.01	-	0 [-.49, .49]	F= 6.22 **p< .001**	F= 1.47 p= .23	Baseline to Post-treatment: p=1.00

	Post-treatment	.69±.02	.29%	.68±.01	−.43%	.23 [-.26, .73]			Baseline to Follow-up: **p=.03**
	Follow-up	.69±.02	.72%	.69±.02	.87%	-.04 [-.54, .45]			
**AD (LH)**	Baseline	1.04±.03	-	1.05±.03	-	-.12 [-.62, .37]	F= 1.10 p= .33	F= 3.02 p= .06	
	Post-treatment	1.04±.03	0%	1.05±.03	−.28%	-.03 [-.52, .46]			
	Follow-up	1.05±.03	.09%	1.06±.02	.66%	-.36 [-.86, .13]			
**AD (RH)**	Baseline	1.07±.03	-	1.08±.03	-	-.34 [-.84, .15]	F= 4.41 **p= .01**	F= 3.09 p= .054	Baseline to Post-treatment: p=1.00 Baseline to Follow-up: p=.12
	Post-treatment	1.08±.03	.65%	1.08±.03	−.27%	-.02 [-.52, .47]			
	Follow-up	1.08±.03	1.21%	1.09±.03	.55%	-.11 [-.61, .38]			
**RD (LH)**	Baseline	.50±.02	-	.50±.02	-	0 [-.49, .49]	F= .83 p= .44	F= 2.08 p= .13	
	Post-treatment	.50±.02	−.59%	.50±.02	.19%	-.16 [-.66, .33]			
	Follow-up	.50±.02	.59%	.50±.03	.39%	.03 [-.46, .53]			
**RD (RH)**	Baseline	.49±.02	-	.48±.02	-	.17 [-.32, .67]	F= 2.93 p= .06	F= .36 p= .69	
	Post-treatment	.49±.03	.20%	.48±.02	−.40%	.28 [-.22, .78]			
	Follow-up	.49±.03	.60%	.49±.03	1.63%	-.06 [-.56, .43]			
Cingulum (cingulate gyrus)									
**FA (LH)**	Baseline	.34±.05	-	.35±.05	-	-.07 [-.57, .42]	F= .02 p= .97	F= .34 p= .71	
	Post-treatment	.33±.04	−3.44%	.34±.05	−1.98%	-.17 [-.67, .32]			
	Follow-up	.34±.06	−1.72%	.34±.05	−1.98%	-.04 [-.54, .44]			
**FA (RH)**	Baseline	.30±.05	-	.31±.04	-	-.18 [-.68, .31]	F= .77 p= .46	F= .35 p= .70	
	Post-treatment	.30±.05	−2.91%	.30±.04	−3.14%	-.16 [-.66, .32]			

	Follow-up	.29±.05	−3.55%	.30±.04	−3.14%	-.20 [-.70, -.70]			
**MD (LH)**	Baseline	.735±.02	-	.73±.02	-	-.04 [-.53, .45]	F= 3.02 p= .054	F= 1.27 p= .28	
	Post-treatment	.73±.02	.54%	.73±.02	−.40%	.20 [-.29, .70]			
	Follow-up	.73±.02	.54%	.74±.02	.67%	-.08 [-.58, .41]			
**MD (RH)**	Baseline	.73±.02	-	.73±.02	-	.07 [-.41, .57]	F= 3.16 p= .50	F= 1.29 p= .28	
	Post-treatment	.74±.02	.68%	.73±.02	.13%	.23 [-.26, .73]			
	Follow-up	.73±.02	.54%	.73±.02	.81%	0 [-.49, .49]			
**AD (LH)**	baseline	1.03±.04	-	1.03±.04	-	-.08 [-.58, .41]	F= .74 p= .48	F= .10 p= .90	
	Post-treatment	1.02±.03	−.67%	1.02±.04	−.96%	-.02 [-.52, .47]			
	Follow-up	1.03±.04	−.28%	1.03±.05	0%	-.15 [-.65, .34]			
**AD (RH)**	Baseline	.99±.04	-	.99±.03	-	-.16 [-.66, .33]	F= .61 p= .54	F= .22 p= .79	
	Post-treatment	.99±.04	−.10%	.99±.02	−.80%	.02 [-.47, .52]			
	Follow-up	.98±.03	−.50%	.99±.03	−.20%	-.26 [-.76, .23]			
**RD (LH)**	Baseline	.58±.04	-	.58±.04	-	.02 [-.47, .52]	F= 1.51 p= .22	F= 1.10 p= .33	
	Post-treatment	.59±.04	1.53%	.58±.04	.17%	.20 [-.28, .70]			
	Follow-up	.59±.05	1.70%	.59±.04	1.54%	.04 [-.45, .53]			
**RD (RH)**	Baseline	.60±.04	-	.60±.04	-	.12 [-.37, .61]	F= 3.60 **p= .03**	F= 1.02 p= .36	Baseline to Post-treatment: p=1.00 Baseline to Follow-up: **p=.04**
	Post-treatment	.61±.04	1.48%	.60±.03	.33%	.29 [-.20, .79]			
	Follow-up	.61±.04	1.98%	.61±.03	1.83%	.14 [-.28, .57]			
Cingulum hippocampus									

**FA (LH)**	Baseline	.24±.03	-	.24±.03	-	0 [-.42, .42]	F= 3.73 **p= .03**	F= 2.13 p= .12	Baseline to Post-treatment: p=1.00 Baseline to Follow-up: **p=.04**
	Post-treatment	.24±.03	0%	.25±.02	2.02%	-.17 [-.60, .24]			
	Follow-up	.26±.02	5.26%	.25±.02	1.61%	.34 [-.08, .77]			
**FA (RH)**	Baseline	.21±.04	-	.22±.03	-	-.07 [-.50, .35]	F= 1.43 p= .24	F= .52 p= .59	
	Post-treatment	.21±.03	−1.36%	.21±.03	−2.70%	0 [-.42, .42]			
	Follow-up	.22±.04	4.56%	.21±.03	−3.15%	.33 [-.09, .76]			
**MD (LH)**	Baseline	.84±.08	-	.85±.06	-	-.06 [-.49, .35]	F= 4.20 **p= .02**	F= 1.51 p= .22	Baseline to Post-treatment: p=.99 Baseline to Follow-up: **p=.01**
	Post-treatment	.84±.09	−.82%	.85±.05	−.35%	-.11 [-.54, .30]			
	Follow-up	.81±.05	−3.77%	.84±.06	−.46%	-.54 [-.97, -.10]			
**MD (RH)**	Baseline	.87±.12	-	.84±.07	-	.22 [-.19, .65]	F= .2 p= .78	F= 1.72 p= .18	
	Post-treatment	.84±.09	−2.52%	.85±.09	.70%	-.04 [-.47, .38]			
	Follow-up	.84±.08	−3.55%	.84±.08	.23%	-.10 [-.53, .32]			
**AD (LH)**	Baseline	1.06±.07	-	1.06±.05	-	-.05 [-.48, .37]	F= 2.22 p=.11	F= .25 p= .77	
	Post-treatment	1.05±.09	−.66%	1.06±.05	.37%	-.18 [-.60, .24]			
	Follow-up	1.03±.07	−2.07%	1.05±.07	−.75%	-.23 [-.66, .19]			
**AD (RH)**	Baseline	1.05±.12	-	1.03±.06	-	.24 [-.18, .67]	F= .97 p= .34	F= 1.16 p= .31	
	Post-treatment	1.02±.09	−2.46%	1.03±.09	.19%	-.04 [-.46, .38]			
	Follow-up	1.03±.07	−2.08%	1.02±.06	−.67%	.12 [-.30, .55]			
**RD (LH)**	Baseline	.74±.08	-	.74±.07	-	-.08 [-.51, .33]	F= 4.23 **p= .01**	F= .88 p= .41	Baseline to Post-treatment: p=1.00

	Post-treatment	.73±.09	−.94%	.74±.06	−1.06%	.07 [-.35, .50]			Baseline to Follow-up: **p=.01**
	Follow-up	.71±.06	−4.31%	.73±.07	−1.73%	-.36 [-.79, .06]			
**RD (RH)**	Baseline	.77±.13	-	.75±.08	-	.20 [-.21, .63]	F= .15 p= .86	F= 1.54 p= .22	
	Post-treatment	.75±.09	−2.56%	.76±.09	.92%	-.03 [-.45, .39]			
	Follow-up	.74±.09	−3.85%	.75±.09	.13%	-.07 [-.50, .35]			
Retrolenticular part of the internal capsule									
**FA (LH)**	Baseline	.47±.02	-	.48±.02	-	-.22 [-.65, .20]	F= 2.15 p= .12	F= 1.96 p= .15	
	Post-treatment	.48±.02	1.25%	.48±.03	−1.03%	.17 [-.25, .60]			
	Follow-up	.48±.02	1.67%	.48±.02	.61%	-.03 [-.46, .39]			
**FA (RH)**	Baseline	.48±.03	-	.47±.02	-	.30 [-.12, .73]	F= .11 p= .89	F= 3.13 p= .053	
	Post-treatment	.48±.02	−1.23%	.48±.03	1.67%	-.16 [-.59, .26]			
	Follow-up	.48±.02	0%	.47±.03	.20%	.26 [-.16, .69]			
**MD (LH)**	Baseline	.74±.03	-	.73±.03	-	.08 [.33, .51]	F= .29 p= .74	F= .64 p= .52	
	Post-treatment	.73±.02	.13%	.74±.03	1.22%	-.21 [-.63, .21]			
	Follow-up	.73±.03	−.67%	.73±.03	.13%	-.09 [-.52, .33]			
**MD (RH)**	Baseline	.71±.04	-	.71±.02	-	.08 [-.34, .51]	F= 3.62 **p= .03**	F= .17 p= .84	Baseline to Post-treatment: p=1.00 Baseline to Follow-up: p=.20
	Post-treatment	.71±.03	−.27%	.71±.02	−.28%	.10 [-.32, .53]			
	Follow-up	.71±.02	.13%	.71±.02	.84%	-.09 [-.51, .33]			
**AD (LH)**	Baseline	1.25±.04	-	1.15±.03	-	2.51 [1.94, 3.09]	F= .01 p= .98	F= .01 p= .99	
	Post-treatment	1.16±.04	−7.48%	1.16±.02	.60%	-.05 [-48, .37]			

	Follow-up	1.16±.05	−7.64%	1.16±.03	.43%	-.04 [-.47, .38]			
	Baseline	1.12±.05	-	1.11±.03	-	.25 [-.17, .68]			
**AD (RH)**	Post-treatment	1.12±.05	−.53%	1.12±.04	.53%	-.02 [-.44, .40]	F= 5.45 **p= .007**	F= .94 p= .39	Baseline to Post-treatment: p=1.00 Baseline to Follow-up: **p=.03**
	Follow-up	1.13±.04	.35%	1.12±.03	1.07%	.07 [-.35, .50]			
	Baseline	.53±.03	-	.52±.04	-	.12 [-.30, .55]			
**RD (LH)**	Post-treatment	.52±.02	−.93%	.53±.04	1.89%	-.25 [-.68, .17]	F= .68 p= .50	F= 1.78 p= .17	
	Follow-up	.52±.03	−1.50%	.52±.03	−.37%	-.02 [-.45, .39]			
	Baseline	.50±.04	-	.51±.03	-	-.04 [-.47, .37]			
**RD (RH)**	Post-treatment	.51±.02	.19%	.50±.03	−1.36%	.19 [-.22, .62]	F= 2.44 p= .09	F= .93 p= .39	
	Follow-up	.51±.02	.19%	.51±.03	1.17%	-.24 [-.67, .18]			
Superior cerebellar peduncle									
	Baseline	.40±.05	-	.38±.04	-	.37 [-.06, .80]			
**FA (LH)**	Post-treatment	.39±.04	2.70%	.39±.04	2.31%	-.04 [-.47, .38]	F= 1.29 p= .28	F= 3.14 p= .053	
	Follow-up	.41±.04	1.97%	.39±.05	2.31%	.34 [-.09, .77]			
	Baseline	.40±.04	-	.38±.04	-	1.37 [.90, 1.85]			
**FA (RH)**	Post-treatment	.39±.04	−2.73%	.38±.04	−1.80%	.21 [-.20, .64]	F= .34 p= .71	F= .34 p= .31	
	Follow-up	.40±.04	.99%	.38±.04	−.51%	.42 [-.00, .86]			
	Baseline	1.06±.15	-	1.08±.15	-	-.13 [-.56, .29]			
**MD (LH)**	Post-treatment	1.08±.14	1.87%	1.10±.14	1.65%	-.128 [-.55, .90]	F= 3.38 **p= .04**	F=1.10 p= .33	Baseline to Post-treatment: p=1.00 Baseline to Follow-up: **p=.04**
	Follow-up	1.05±.13	.65%	1.04±.11	−3.31%	.06 [-.36, .49]			

**MD (RH)**	Baseline	1.02±.13	-	1.03±.14	-	-.04 [-.47, .37]	F= .34 p= .70	F= 1.61 p= .20	
	Post-treatment	1.04±.12	1.26%	1.08±.15	4.92%	-.32 [-.75, .10]			
	Follow-up	1.04±.14	1.36%	1.04±.12	.67%	0 [-.42, .42]			
**AD (LH)**	Baseline	1.52±.14	-	1.52±.15	-	-.006 [-.43, .42]	F= 2.90 p= .06	F= 2.43 p= .09	
	Post-treatment	1.53±.14	.65%	1.56±.13	1.17%	-.20 [-.63, .22]			
	Follow-up	1.52±.13	0%	1.49±.11	−2.48%	.28 [-.14, .71]			
**AD (RH)**	Baseline	1.47±.13	-	1.46±.14	-	.09 [-.33, .20]	F= .32 p=.72	F= 2.70 p= .07	
	Post-treatment	1.48±.12	.68%	1.52±.13	4.03%	-.25 [-.68, .17]			
	Follow-up	1.49±.14	1.70%	1.46±.13	.34%	.20 [-.22, .63]			
**RD (LH)**	Baseline	.83±.16	-	.86±.15	-	-.18 [-.61, .24]	F= 3.46 **p= .03**	F= 1.12 p= .33	Baseline to Post-treatment: p=.73 Baseline to Follow-up: **p=.03**
	Post-treatment	.85±.15	2.76%	.87±.15	.92%	-.09 [-.52, .33]			
	Follow-up	.82±.13	−1.20%	.82±.13	−4.86%	.01 [-.41, .44]			
**RD (RH)**	Baseline	.80±.14	-	.82±.15	-	-.11 [-.54, .31]	F= .39 p= .67	F= 1.09 p= .34	
	Post-treatment	.82±.13	2.35%	.87±.16	5.83%	-.31 [-.74, .12]			
	Follow-up	.81±.15	.99%	.82±.12	.48%	-.09 [-.52, .33]			
Superior longitudinal fasciculus									
**FA (LH)**	Baseline	.30±.03	-	.30±.03	-	0 [-.43, .43]	F= .46 p= .63	F= 1.19 p= .31	
	Post-treatment	.29±.03	−1.96%	.30±.02	.32%	-.23 [-.66, .20]			
	Follow-up	.31±.02	1.63%	.30±.03	−.32%	.221 [-.21, .65]			
**FA (RH)**	Baseline	.32±.03	-	.32±.03	-	0 [-.43, .43]	F= .60 p= .54	F= .83 p= .44	

	Post-treatment	.32±.02	−.92%	.32±.02	.30%	-.15 [-.58, .27]			
	Follow-up	.32±.03	1.23%	.32±.03	.61%	.06 [-.36, .49]			
**MD (LH)**	Baseline	.74±.03	-	.74±.03	-	.05 [-.37, .48]	F= .93 p= 3.9	F= 1.24 p= .29	
	Post-treatment	.74±.03	.67%	.74±.03	−.13%	.22 [-.20, .66]			
	Follow-up	.74±.03	−.53%	.80±.03		-.23 [-.67, .19]			
**MD (RH)**	Baseline	.73±.04	-	.73±.03	-	0 [-.43, .43]	F= 2.83 p= .06	F= 2.13 p= .12	
	Post-treatment	.74±.03	.27%	.73±.03	−.40%	.15 [-.28, .58]			
	Follow-up	.74±.04	.94%	.74±.03	.81%	.02 [-.40, .45]			
**AD (LH)**	Baseline	.97±.02	-	.97±.02	-	.09 [-.33, .52]	F= 1.28 p=.28	F= 1.40 p= .245	
	Post-treatment	.98±.02	1.12%	.97±.02	.10%	.48 [.043, .91]			
	Follow-up	.97±.02	0%	.98±.02	.71%	-.21 [-.64, .21]			
**AD (RH)**	Baseline	.98±.02	-	.98±.02	-	.08 [-.34, .51]	F= 18.28 **p < 0.001**	F= 1.97 p=.15	Baseline to Post-treatment: p=1.00 Baseline to Follow-up: **p< .001**
	Post-treatment	.98±.02	0%	.98±.02	−.2%	.14 [-.28, .57]			
	Follow-up	.99±.02	1.21%	.99±.02	1.22%	.07 [-.35, .50]			
**RD (LH)**	Baseline	.62±.04	-	.62±.03	-	.02 [-.40, .45]	F= 1.13 p= .32	F= 1.15 p= .32	
	Post-treatment	.63±.04	1.27%	.62±.04	−.31%	.25 [-.17, .68]			
	Follow-up	.62±.04	.31%	.63±.04	.95%	-.16 [-.59, .26]			
**RD (RH)**	Baseline	.61±.04	-	.61±.03	-	.15 [-.28, .58]	F= 1.40 p= .25	F= 1.89 p= .16	
	Post-treatment	.62±.05	.32%	.62±.04	1.30%	0 [-.43, .43]			
	Follow-up	.61±.04	−.16%	.61±.04	.65%	.021			

						[-.40, .45]		
Tapetum								
**FA (LH)**	Baseline	.34±.08	-	.34±.06	-	-.05<br>[-.48, .37]	F= .33<br>p= .71	F= .53<br>p= .58
	Post-treatment	.35±.06	2.91%	.348±.06	.28%	.07<br>[-.35, .50]		
	Follow-up	.35±.07	3.20%	.36±.05	4.03%	-.12<br>[-.55, .30]		
**FA (RH)**	Baseline	.33±.07	-	.35±.05	-	-.28<br>[-.71, .14]	F= 1.00<br>p= .37	F= 1.79<br>p= .17
	Post-treatment	.34±.07	2.66%	.34±.06	−2.51%	-.02<br>[-.45, .40]		
	Follow-up	.35±.06	6.21%	.36±.05	2.79%	-.53<br>[-.97, -.09]		
**MD (LH)**	Baseline	1.44±.38	-	1.03±.31	-	1.17<br>[.70, .70]	F= .55<br>p= .58	F= .63<br>p= .53
	Post-treatment	1.39±.31	−3.59%	1.41±.31	36.70%	-.07<br>[-.50, .35]		
	Follow-up	1.40±.33	−3.17%	1.33±.28	28.51%	.21<br>[-.21, .64]		
**MD (RH)**	Baseline	1.44±.34	-	1.29±.27	-	.46<br>[.02, .902]	F= .66<br>p= .51	F= 2.27<br>p= .11
	Post-treatment	1.42±.30	−1.04%	1.33±.30	3.16%	.29<br>[-.14, .72]		
	Follow-up	1.36±.28	−4.99%	1.29±.26	.15%	.25<br>[-.17, .6]		
**AD (LH)**	Baseline	1.90±.36	-	1.84±.31	-	.18<br>[-.24, .62]	F= .43<br>p=.64	F= .61<br>p= .54
	Post-treatment	1.85±.31	−2.62%	1.88±.29	2.06%	-.07<br>[-.50, .351]		
	Follow-up	1.86±.31	−2.15%	1.80±.28	−2.28%	.21<br>[-.21, .71]		
**AD (RH)**	Baseline	1.87±.32	-	1.73±.27	-	.46<br>[.02, .90]	F= .44<br>p= .64	F= 2.10<br>p= .13
	Post-treatment	1.87±.27	−.10%	1.82±.29	4.95%	.19<br>[-.23, .62]		
	Follow-up	1.82±.26	−2.82%	1.74±.26	.4%	.30<br>[-.12, .73]		
**RD (LH)**	Baseline	1.21±.39	-	1.16±.31	-	.16	F= .51	F= .56

						[-.26, .59]	p= .60	p= .57
	Post-treatment	1.16±.32	−4.34%	1.88±.32	61.92%	-.09<br>[-.52, .33]		
	Follow-up	1.16±.34	−4.67%	1.10±.32	−4.82%	.16<br>[-.26, .59]		
**RD (RH)**	Baseline	1.22±.35	-	1.07±.28	-	.67<br>[.23, 1.11]	F= .43<br>p= .51	F= .99<br>p= .10
	Post-treatment	1.20±.31	−1.63%	1.15±.11	7.33%	.19<br>[-.23, .62]		
	Follow-up	1.14±.29	−6.62%	1.06±.26	−.74%	.26<br>[-.16, .69]		

All analyses were performed using Linear Mixed Models. When a variable was assessed for more than two times ‘repeated covariance type’ was set at ‘unstructured’.
Age was entered as covariate.
Significant p-values were printed in **bold**.
MD, AD, and RD are in units of 10^{-3} $mm^2/_{sec}$.
Abbreviations: WM= white matter, DTI= diffusion tensor imaging FA= fractional anisotropy, MD= mean diffusivity, AD= axial diffusivity, and RD= radial diffusivity, LH= left hemisphere, RH= right hemisphere. Follow-up = 12 months after start treatment.
